# The Effects of Amphiregulin Induced MMP-13 Production in Human Osteoarthritis Synovial Fibroblast

**DOI:** 10.1155/2014/759028

**Published:** 2014-07-24

**Authors:** Yi-Te Chen, Chun-Han Hou, Sheng-Mou Hou, Ju-Fang Liu

**Affiliations:** ^1^Department of Orthopedic Surgery, Shin Kong Wu Ho-Su Memorial Hospital, No. 95, Wen-chang Road, Shi-lin District, Taipei 11101, Taiwan; ^2^Department of Orthopedic Surgery, National Taiwan University Hospital, No.1, Section 1, Ren-ai Road, Zhong-zheng District, Taipei 10051, Taiwan; ^3^Central Laboratory, Shin-Kong Wu Ho-Su Memorial Hospital, No. 95, Wenchang Road, Shi-lin District, Taipei 11101, Taiwan

## Abstract

Osteoarthritis (OA) belongs to a group of degenerative diseases. Synovial inflammation, cartilage abrasion, and subchondral sclerosis are characteristics of OA. Researchers do not fully understand the exact etiology of OA. However, matrix metalloproteinases (MMPs), which are responsible for cartilage matrix degradation, play a pivotal role in the progression of OA. Amphiregulin (AREG) binds to the EGF receptor (EGFR) and activates downstream proteins. AREG is involved in a variety of pathological processes, such as the development of tumors, inflammatory diseases, and rheumatoid arthritis. However, the relationship between AREG and MMP-13 in OA synovial fibroblasts (SFs) remains unclear. We investigated the signaling pathway involved in AREG-induced MMP-13 production in SFs. AREG caused MMP-13 production in a concentration- and time-dependent manner. The results of using pharmacological inhibitors and EGFR siRNA to block EGFR revealed that the EGFR receptor was involved in the AREG-mediated upregulation of MMP-13. AREG-mediated MMP-13 production was attenuated by PI3K and Akt inhibitors. The stimulation of cells by using AREG activated p65 phosphorylation and p65 translocation from the cytosol to the nucleus. Our results provide evidence that AREG acts through the EGFR and activates PI3K, Akt, and finally NF-kappaB on the MMP-13 promoter, thus contributing to cartilage destruction during osteoarthritis.

## 1. Introduction

Osteoarthritis (OA), the most common joint disease, belongs to a group of degenerative diseases that often occur in middle-aged and older people. Synovial inflammation, cartilage surface abrasion, subchondral sclerosis, and osteophyte formation are characteristics of OA [1]. The current goals of OA therapy are the control of pain and improvement of joint function. However, these therapeutic strategies cannot reverse damage to joint tissues or halt the progression of OA, and surgical intervention is unavoidable. OA is regarded as a complex disease that can be triggered by numerous factors, such as obesity, inflammation, aging, genetic factors, and injury [2]. Previous studies have discovered that inflammatory cytokines and matrix metalloproteinases (MMPs) play a critical role in the progression of OA. Patients with OA exhibited increased levels of Aggrecan fragments and MMPs in the synovial fluid [3]. Recently, several studies have demonstrated that MMPs are produced by chondrocytes and synovium during the development of cartilage degradation [4, 5]. Therefore, researchers have developed inhibitors of the enzyme activity of MMPs as a therapeutic target in OA.

MMPs, a large family of structurally related zinc-dependent proteases, are involved in the degradation of the extracellular matrix. MMPs can be divided into 4 main subtypes: collagenases (MMP-1, MMP-8, and MMP-13), stromelysins (MMP-3, MMP-10, and MMP-11), gelatinases (MMP-2 and MMP-9), and membrane-type matrix metalloproteinases (MT-MMP, MMP-14, MMP-15, MMP-16, MMP-17, MMP-24, and MMP-25) [6]. Chondrocytes, synovial-fibroblast cells, neutrophils, and macrophages can synthesize and secrete MMPs. IL-1*β* and TNF-*α* stimulate MMPs, causing cartilage matrix degradation [7, 8]. MMP-13 (also known as collagenase-3) is an essential enzyme in types I, II, III, IV, IX, X, and XIV collagen, and the proteoglycan degradation of cartilage and the expression of MMP-13 are considerably higher in patients with OA [9]. A complete understanding of the various factors and pathways involved in the regulation of MMP expression could be useful for the development of potential therapies.

Amphiregulin (AREG), a member of the epidermal growth factor (EGF) family, binds to the EGF receptor (EGFR) and activates downstream signaling pathways in an autocrine, paracrine, and juxtacrine manner [10]. AREG plays a critical role in several biological processes, including cell growth, cell proliferation, cell migration, nerve generation, and bone formation [11]. Overexpression of AREG can also indicate several diseases such as cancer, pulmonary fibrosis, arthritis, and asthma [12]. AREG induces EGFR dimerization, autophosphorylation, and tyrosine phosphorylation of downstream proteins, which consequently activates 2 major pathways, MEK/ERK and PI3K/Akt. Recent studies have demonstrated that activated EGFR and PI3K/Akt pathways mediate MMP expression in human keratinocytes and various tumors cells [13]. In addition, ERK and PI3K/Akt pathways in human lens epithelial cells mediate EGF-induced cell migration and MMP-2 expression [13].

One previous study demonstrated that AREG stimulates the production and overexpression of proinflammatory cytokines in the synovium, synovial fluid, and cartilage of patients with rheumatoid arthritis (RA) [14]. In addition, the overexpression of AREG may promote synovial hyperplasia through MMP-13 in patients with RA [14]. However, researchers do not yet completely understand the relationship between AREG in synovial fibroblasts and OA progression. Therefore, we explored the signaling pathways involved in AREG-induced MMP-13 production in human OA synovial fibroblasts (OASFs), as well as the role that AREG plays in the pathogenesis of OA, to determine whether AREG is an appropriate target for drug intervention in OA in the future.

## 2. Materials and Methods

### 2.1. Materials

Dulbecco's modified Eagle's medium (DMEM), fetal bovine serum (FBS), Lipofectamine 2000, and TRIzol were purchased from Invitrogen (Carlsbad, CA, USA). Cell culture dishes, 6-well plates, and 12-well plates were purchased from Greiner Bio-One (Frickenhausen, Germany). Polyvinyldifluoride (PVDF) membrane and an Immobilon Western Chemiluminescent HRP substrate detection system were purchased from Millipore (Billerica, MA, USA). Polyclonal antibodies specific for EGFR, PI3K, Akt, IKK*α*/*β*, I*κ*B, p65, and *β*-actin were purchased from Santa Cruz Biotechnology (Santa Cruz, CA, USA). Polyclonal rabbit antibodies specific for EGFR phosphorylated at Tyr^992^ and Tyr^1068^, PI3K phosphorylated at Tyr^458/199^, Akt phosphorylated at ser^473^, IKK*α*/*β* phosphorylated at ser^176/180^, I*κ*B*α* phosphorylated at ser^32/36^, and p65 phosphorylated at ser^536^ were purchased from Cell Signaling and Neuroscience (Danvers, MA, USA). 4-[(3-Bromophenyl) amino]-6-(methylamino)-pyrido[3,4-d]pyrimidine (PD158780), N8-(3-Chloro-4-fluorophenyl)-N2-(1-methylpiperidin-4-yl)-pyrimido[5,4-d]pyrimidine-2,8-diamine (BIBX1382), LY294002, Wortmannin, 1L-6-hydroxymethyl-chiro-inositol-2-((R)-2-O-methyl-3-O-octadecyl carbonate) (Akti), pyrrolidine dithiocarbamate (PDTC), and L-1-tosylamido-2-phenylenylethyl chloromethyl ketone (TPCK) were purchased from Calbiochem (San Diego, CA, USA). Recombinant human AREG was purchased from PeproTech (Rocky Hill, NJ, USA). Small interfering RNA (siRNA) of EGFR and control was purchased from Santa Cruz Biotechnology (Santa Cruz, CA, USA). Nuclear factor kappa B (NF-*κ*B) luciferase plasmid was purchased from Stratagene (La Jolla, CA, USA). p85 and Akt1 (Akt1 K179A) dominant-negative mutants were provided by Dr. Fu (National Taiwan University, Taipei, Taiwan). A pSV-*β*-galactosidase vector and luciferase assay kit were purchased from Promega (Madison, MA, USA). All other chemicals were purchased from Sigma-Aldrich (St. Louis, MO, USA).

### 2.2. Cell Cultures

Written informed consent was obtained from all patients and the study was approved by the Institutional Review Board of Shin Kong Wu Ho-Su Memorial Hospital. Human synovial fibroblasts were isolated by subjecting synovial tissue samples obtained from patients with OA during knee-replacement surgeries to collagenase treatment, and samples of nonarthritic synovial tissues were obtained during arthroscopy after trauma or joint derangement. Fresh synovial tissues were minced, digested, isolated, and maintained using the methods outlined in previous studies [15–17]. Four independent experiments were performed.

### 2.3. RNA Extraction and Quantitative Real-Time Polymerase Chain Reaction

Total RNA was extracted from cells by using a TRIzol kit; then 2 *μ*g of RNA was used to synthesize complementary DNA (cDNA) by using reverse transcriptase (Invitrogen, Carlsbad, CA, USA). Quantitative real-time polymerase chain reaction (qPCR) was conducted using SYBR Green (KAPA Biosystems, Woburn, MA, USA) according to the manufacturer protocol, and reactions were performed using a StepOnePlus machine (Applied Biosystems, Foster City, CA, USA). The reaction conditions were 10 min at 95°C for polymerase activation and 40 cycles of 15 s at 95°C and 60 s at 60°C. Human MMP-1, MMP-2, MMP-3, MMP-7, MMP-9, MMP-12, MMP-13, and glyceraldehyde 3-phosphate dehydrogenase (GAPDH), purchased from Sigma-Aldrich (St. Louis, MO, USA), were used as primers to amplify target genes. The expression levels of MMPs were determined by normalizing them to that of GAPDH. The threshold cycle (Ct) was set above the nontemplate control background and within the linear phase of target gene amplification to calculate the cycle numbers at which the transcript was detected (denoted Ct). Each sample was assayed in triplicate and the data displayed represent 3 independent experiments.

### 2.4. Western Blot Analysis

Cellular lysates were prepared using the methods outlined in previous studies [18, 19]. Proteins were resolved on sodium dodecyl sulfate (SDS) polyacrylamide gel electrophoresis gel and transferred to Immobilon PVDF membranes. The blots were blocked with 5% BSA for 1 h at room temperature and then probed with rabbit antihuman antibodies against EGFR, PI3K, Akt, IKK*α*/*β*, I*κ*B, and p65 (1 : 1000) for 1 h at room temperature. After 3 washes, the blots were subsequently incubated with donkey anti-rabbit peroxidase-conjugated secondary antibody (1 : 3000) for 1 h at room temperature. The blots were visualized using enhanced chemiluminescence and FUJI Super RX-N X-RAY film (Fujifilm Corporation, Tokyo, Japan). Quantitative data were obtained using TotalLab 2.0 software (Nonlinear Dynamics, BioSystematica, UK).

### 2.5. Zymography Analysis

Zymography was used to investigate the possible mechanism of AREG that induces MMP-13 by detecting MMP-13 expression levels in culture mediums. In brief, supernatants from OASF cultures were mixed with sample buffer without the use of a reducing agent or heat, followed by electrophoresis. Afterward, the gel was washed using 2.5% Triton X-100 to remove SDS, rinsed with 50 mM Tris-HCl, pH 7.5, and then incubated overnight at room temperature with a developing buffer (50 mM Tris-HCl, pH 7.5, 5 mM CaCl_2_, 1 mM ZnCl_2_, 0.02% thimerosal, and 1% Triton X-100). Zymographic activity was analyzed by staining the samples with 1% Coomassie Blue [20].

### 2.6. Measurement of NF-*κ*B Promoter-Luciferase Activity

NF-*κ*B promoter-luciferase activity was measured using the methods outlined in previous studies [21]. In brief, cells were cotransfected with 0.8 *μ*g of luciferase plasmid and 0.4 *μ*g of *β*-galactosidase expression vector. Cells were grown to 70% confluence in 12-well plates and were transfected with Lipofectamine 2000 (LF2000; Invitrogen) the following day. DNA and LF2000 were premixed and then applied to the cells. After 24 h of transfection, cells were then incubated with the indicated agents. After 24 h of incubation, fresh luciferase assay buffer (80 *μ*L) was added to 20 *μ*L of cell lysates (Promega, Madison, WI, USA) and luciferase activity was measured using a microplate luminometer. Luciferase activity was normalized to transfection efficiency based on the cotransfected *β*-galactosidase expression vector.

### 2.7. Immunofluorescence Staining

Cells were grown on 12 mm coverslips. After treatment using the indicated agents, cells were fixed with 4% paraformaldehyde at room temperature. Thirty minutes later, 5% nonfat milk in phosphate buffer saline (PBS) containing 0.25% Triton X-100 was added to the cells. The cells were then incubated in rabbit anti-p65 (1 : 100) and fluorescein isothiocyanate- (FITC-) conjugated goat anti-rabbit secondary antibodies (1 : 500; Leinco Technology Inc., St. Louis, MO, USA) for 1 h. FITC was detected using a Zeiss fluorescence microscope [20].

### 2.8. Statistics

Values are reported as means ± standard error of the mean. A statistical comparison of the 2 samples was performed using Student's *t*-test. Statistical comparisons of more than 2 groups were performed using one-way analysis of variance (ANOVA) with the Bonferroni post hoc test. In all comparisons, *P* < 0.05 was considered significant.

## 3. Results

### 3.1. AREG-Induced MMP-13 Production in Human Synovial Fibroblasts

**Figure 1:**
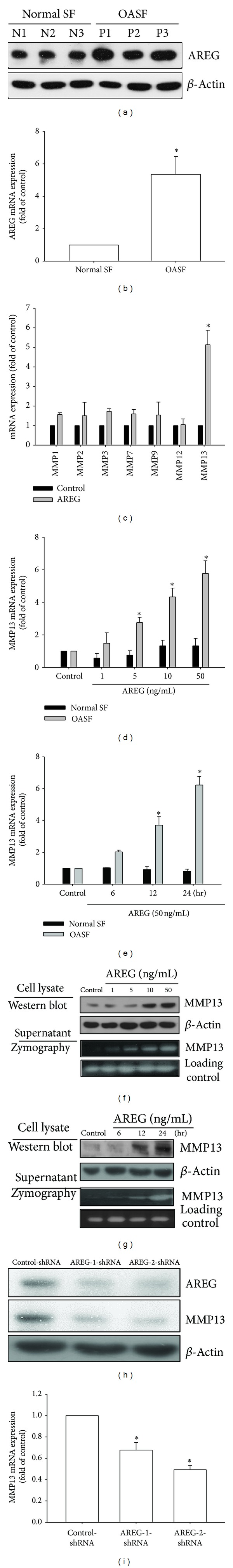
Concentration- and time-dependent increases in MMP-13 production by AREG. ((a) and (b)) Human synovial fibroblasts were obtained from normal (*n* = 3) or osteoarthritis patients (*n* = 3) and examined by Western blot and qPCR for the expression of AREG. (c) OASF and normal SF were incubated with AREG for 24 h. The mRNA expression of MMPs was examined by qPCR (*n* = 4). ((d) and (e)) OASF and normal SF were incubated with various concentrations of AREG for 24 h or with AREG (50 ng/mL) for 6, 12, or 24 h. The mRNA expression of MMP-13 was examined by qPCR (*n* = 4). ((f) and (g)) OASFs were incubated with various concentrations of AREG for 24 h or with AREG (50 ng/mL) for 6, 12, or 24 h, and supernatants and cell lysates were then collected. MMP-13 protein levels in cell lysates were determined by Western blot analysis. MMP-13 enzymatic activity in cell lysates and supernatants was measured using zymography. Both protein levels and enzymatic activity increased in a time-dependent manner (*n* = 4). (h) The protein and mRNA levels of AREG and MMP-13 in control-shRNA and AREG-shRNA OASF were examined by Western blot and qPCR. Results are expressed as the mean ± SEM. *P < 0.05 compared with control; ^#^ *P* < 0.05 compared with AREG-treated group.

AREG plays a critical role during RA pathogenesis [14]. However, the role of AREG in OA is unclear. Therefore, we first compared the AREG levels of normal human synovial fibroblast (normal SF) and OASF. The Western blot and qPCR results displayed in Figures 1(a) and 1(b) indicate that the protein levels and mRNA expression of AREG were higher in the OASFs than in the normal SFs. Previous research has indicated that MMP-1, MMP-2, MMP-3, MMP-7, MMP-9, MMP-12, and MMP-13 are expressed in high quantities in the cells of patients with OA [22]. Another study demonstrated that AREG induces MMP expression in various cell types [23]. Therefore, we hypothesized that any of these MMPs could be involved in AREG-directed OA pathogenesis. We used real-time PCR to detect levels of mRNA expression of MMPs in OASFs. The expression of MMP-13 was significantly higher than that of other MMPs (Figure 1(c)). Additionally, we discovered that AREG induced MMP-13 production in a concentration-dependent manner (Figures 1(d) and 1(f)), and induction occurred in a time-dependent manner in OASFs (Figures 1(e) and 1(g)). By contrast, AREG did not affect MMP-13 expression in normal SFs (Figures 1(d) and 1(e)). Therefore, OASFs are more sensitive to AREG than are normal SFs. To investigate AREG-mediated MMP-13 expression in OASFs further, we established AREG-shRNA expression cells. We used Western blot to compare the level of AREG expression in stable transfectants. Expression of AREG was drastically inhibited in OASF/AREG-shRNA cells (Figure 1(h)). In addition, knockdown of AREG also downregulated the expression of MMP-13 in OASFs (Figures 1(h) and 1(i)). These results indicated that AREG increased MMP-13 production in human OASFs.

### 3.2. AREG/EGFR Interaction Caused the Expression of MMP-13 in OASF

**Figure 2:**
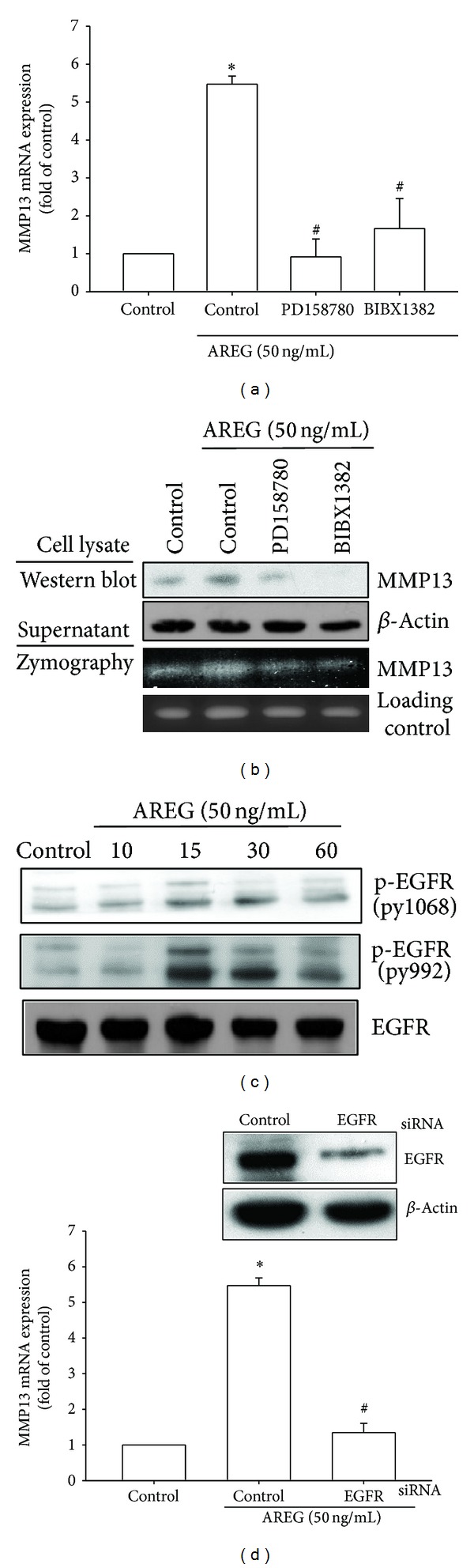
EGFR is involved in AREG-mediated MMP-13 production in synovial fibroblast. (a) OASFs were pretreated for 30 min with PD158780 (1 *μ*M) and BIBX1382 (1 *μ*M) followed by stimulation with AREG for 24 h, and MMP-13 expression was examined by qPCR. (b) OASFs were pretreated for 30 min with PD158780 (1 *μ*M) and BIBX1382 (1 *μ*M) followed by stimulation with AREG for 24 h, and MMP-13 protein levels in cell lysates were determined by Western blot analysis. MMP-13 enzymatic activity in cell lysates and supernatants was measured using zymography. (c) OASFs were incubated with AREG for indicated time intervals, and EGFR phosphorylation was examined by Western blot. (d) OASFs were transfected for 24 h with EGFR siRNA, followed by stimulation with AREG for 24 h, and MMP-13 expression was examined by qPCR. Results are expressed as the mean ± SEM (*n* = 3). *P < 0.05 compared with control; ^#^ *P* < 0.05 compared with AREG-treated group.

All EGF-like ligands affect cell functions by binding to EGFR [24]. Therefore, we hypothesized that EGFR is involved in AREG-induced MMP-13 production. Pretreating OASFs by using EGFR inhibitors (PD158780 and BIBX1382) [25, 26] for 30 min markedly inhibited AREG-induced MMP-13 mRNA expression (Figure 2(a)). In addition, the results of Western blot and zymographic analysis indicated that EGFR inhibitors reduced MMP-13 expression (Figure 2(b)). Activation of EGFR leads to tyrosine autophosphorylation; therefore, we examined the level of phosphorylated EGFR at tyrosine 1068 and 992 after AREG treatment. The results indicated that AREG treatment increased the level of phosphorylated EGFR (Figure 2(c)). Furthermore, to test whether EGFR caused AREG to increase MMP-13 production in OASFs, we knocked down EGFR expression by transfecting the OASFs with EGFR siRNA and determined that EGFR siRNA inhibited AREG-induced MMP-13 production at the mRNA level (Figure 2(d)). These results suggest that the activation of EGFR could be the cause of AREG-induced MMP-13 expression.

### 3.3. Involvement of the PI3K/Akt Signaling Pathway in AREG-Mediated MMP-13 Production

**Figure 3:**
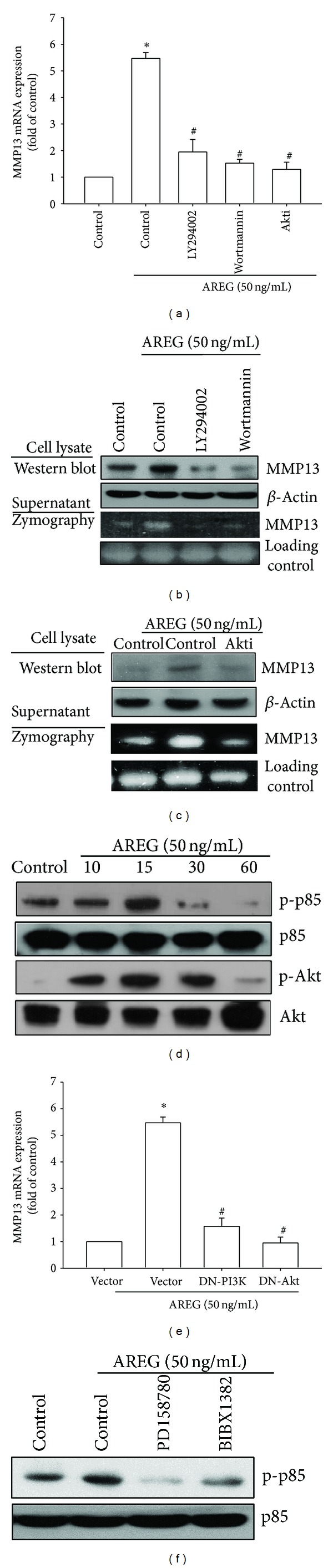
PI3K/Akt is involved in AREG-mediated MMP-13 production in synovial fibroblasts. (a) OASFs were pretreated for 30 min with PI3K inhibitors (LY294002 (5 *μ*M) and Wortmannin (0.5 *μ*M)) and Akt inhibitor (Akti (5 *μ*M)) for 30 min followed by stimulation with AREG for 24 h, and MMP-13 expression was examined by qPCR. ((b) and (c)) OASFs were pretreated for 30 min with PI3K inhibitors (LY294002 (5 *μ*M) and Wortmannin (0.5 *μ*M)) and Akt inhibitor (Akti (5 *μ*M)) for 30 min followed by stimulation with AREG for 24 h; MMP-13 protein levels in cell lysates were determined by Western blot analysis. MMP-13 enzymatic activity in cell lysates and supernatants was measured using zymography. (d) OASFs were incubated with AREG for indicated time intervals, and p85 and Akt phosphorylation was examined by Western blot. (e) OASFs were transfected for 24 h with PI3K and Akt mutant followed by stimulation with AREG for 24 h, and MMP-13 expression was examined by qPCR. (f) OASFs were pretreated for 30 min with PD158780 (1 *μ*M) and BIBX1382 (1 *μ*M) followed by stimulation with AREG for 15 min; MMP-13 protein levels in cell lysates were determined by Western blot analysis. Results are expressed as the mean ± SEM (*n* = 3). *P < 0.05 compared with control; ^#^ *P* < 0.05 compared with AREG-treated group.

Previous research has indicated that the interaction of AREG and EGFR activates several signaling pathways, including PI3K/Akt [13]. Furthermore, one study indicated that PI3K/Akt mediates MMP-13 gene expression in various cell types [27]. Therefore, we investigated whether PI3K and Akt were involved in AREG-induced MMP-13 expression. Figures 3(a), 3(b), and 3(c) indicate that pretreatment with PI3K inhibitors (LY294002 (5 *μ*M) and Wortmannin (0.5 *μ*M)) or Akt inhibitor (Akti (5 *μ*M)) markedly attenuated AREG-induced MMP-13 production. Next, we directly measured p85 and Akt phosphorylation in response to AREG. AREG caused a significant increase in the phosphorylation of p85 and Akt in OASFs (Figure 3(d)). In addition, transfection of OASF with PI3K and Akt mutants reduced AREG-increased MMP-13 production (Figure 3(e)). Therefore, blocking PI3K and Akt can abolish AREG-mediated MMP-13 expression. Finally, the pretreatment of cells with PD158780 and BIBX1382 inhibited the AREG-mediated increase in p85 phosphorylation. These results indicated that AREG induces MMP-13 production through EGFR activation, which consequently activates the PI3K and Akt signaling pathways in OASFs.

### 3.4. Involvement of NF-*κ*B in AREG-Induced MMP-13 Production

**Figure 4:**
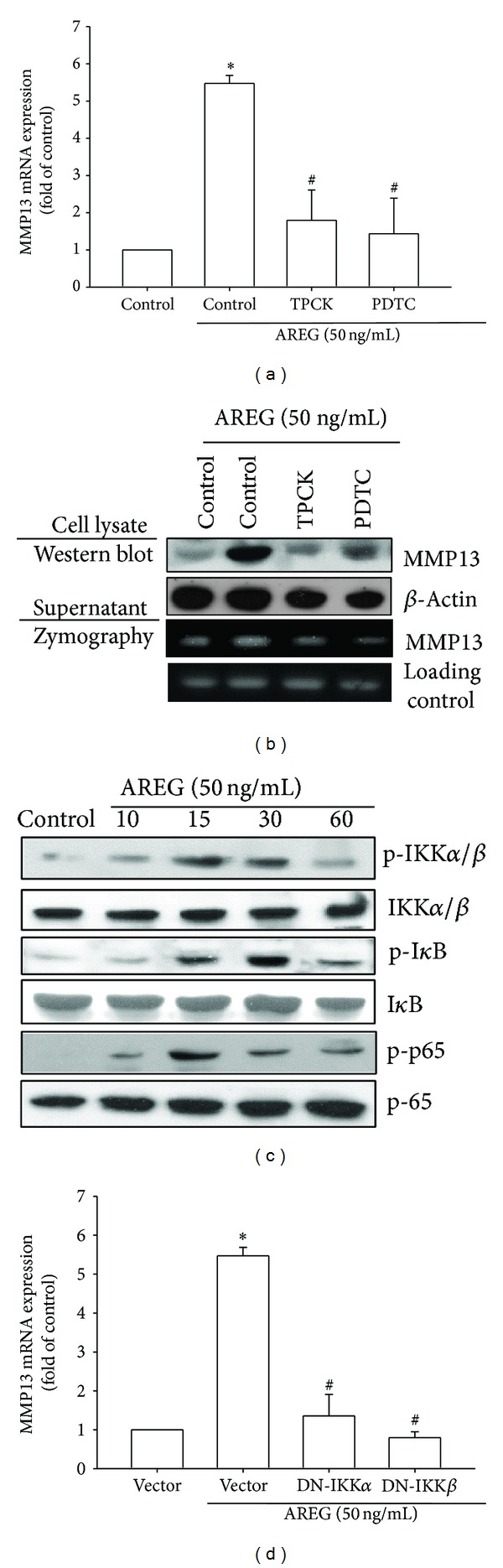
NF-*κ*B is involved in the potentiation of MMP-13 production by AREG. (a) OASFs were pretreated for 30 min with PDTC (5 *μ*M) and TPCK (5 *μ*M) followed by stimulation with AREG for 24 h, and MMP-13 expression was examined by qPCR. (b) OASFs were pretreated for 30 min with PDTC (5 *μ*M) and TPCK (5 *μ*M) for 30 min followed by stimulation with AREG for 24 h; MMP-13 protein levels in cell lysates were determined by Western blot analysis. MMP-13 enzymatic activity in cell lysates and supernatants was measured using zymography. (c) OASFs were incubated with AREG for indicated time intervals; p-IKK*α*/*β*, p-I*κ*B*α*, and p-p65 expression was determined by Western blot analysis. (d) OASFs were transfected for 24 h with IKK*α* and IKK*β* mutant followed by stimulation with AREG for 24 h; and MMP-13 expression was examined by qPCR. Results are expressed as the mean ± SEM (*n* = 3). *P < 0.05 compared with control; ^#^ *P* < 0.05 compared with AREG-treated group.

**Figure 5:**
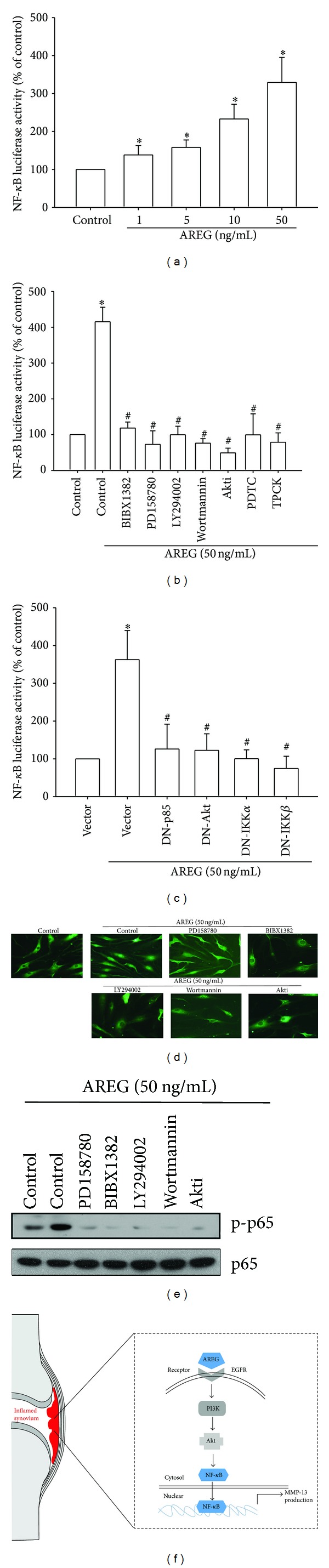
AREG induced NF-*κ*B activation through EGFR/PI3K/Akt pathway. ((a), (b), and (c)) OASFs were incubated with various concentrations of AREG or pretreated with PI3K inhibitors (LY294002 (5 *μ*M) and Wortmannin (0.5 *μ*M)), Akt inhibitor (Akti (5 *μ*M)), or NF-*κ*B inhibitors (PDTC (5 *μ*M), or TPCK (5 *μ*M)) for 30 min or transfected with PI3K, Akt, IKK*α*, and IKK*β* mutant before exposure to AREG. NF-*κ*B luciferase activity was measured, and the results were normalized to the *β*-galactosidase activity. (d) OASFs were pretreated with LY294002, Wortmannin, or Akti for 30 min and then stimulated with AREG for 120 min, and p65 immunofluorescence staining was examined. (e) OASFs were pretreated with LY294002, Wortmannin, or Akti for 30 min followed by stimulation with AREG for 60 min, and p-p65 expression was examined by Western blot analysis. (f) Schematic presentation of the signaling pathways is involved in AREG-induced MMP-13 production in human synovial fibroblast. Results are expressed as the mean ± SEM (*n* = 4). *P < 0.05 compared with control; ^#^ *P* < 0.05 compared with AREG-treated group.

Activation of NF-*κ*B can induce the production of MMP-13 in the cells of patients with RA or OA [28]. NF-*κ*B is a transcriptional activator that plays a vital role in the pathogenesis of OA [29]. To examine whether NF-*κ*B is involved in the signal transduction pathway leading to AREG-induced MMP-13 production, we used NF-*κ*B inhibitors (PDTC) and I*κ*B protease inhibitors (TPCK). The pretreatment of cells with PDTC (5 *μ*M) and TPCK (5 *μ*M) reduced AREG-increased MMP-13 mRNA and protein expression (Figures 4(a) and 4(b)). We further examined the upstream molecules involved in AREG-induced NF-*κ*B activation. The stimulation of OASFs by using AREG increased IKK*α*/*β*, I*κ*B*α*, and p65 phosphorylation in a time-dependent manner (Figure 4(c)). Transfection of cells with IKK*α* and IKK*β* mutant reduced AREG-induced MMP-13 production (Figure 4(d)). To confirm that NF-*κ*B is involved in AREG-induced MMP-13 expression, we performed transient transfection by using NF-*κ*B promoter-luciferase constructs. OASFs incubated with AREG caused NF-*κ*B promoter activity to increase in a dose-dependent manner (Figure 5(a)). The increase in NF-*κ*B activity caused by AREG was antagonized by PI3K inhibitors (LY294002 (5 *μ*M) and Wortmannin (0.5 *μ*M)), Akt inhibitor (Akti (5 *μ*M)), NF-*κ*B inhibitors (PDTC (5 *μ*M) and TPCK (5 *μ*M)), and p85, Akt, IKK*α*, and IKK*β* mutants (Figures 5(b) and 5(c)). Furthermore, LY294002, Wortmannin, and Akti reduced AREG-mediated p65 phosphorylation and translocation into the nucleus (Figures 5(d) and 5(e)). These data suggest that the activation of the EGFR, PI3K, Akt, and NF-*κ*B pathways is required for AREG-induced MMP-13 production in human synovial fibroblasts.

## 4. Discussion

Previous studies have reported that EGF-like ligands, especially AREG, are involved in the pathology of RA. Increasing evidence suggests that AREG plays a vital role in the progression of joint degenerative disorders [14, 30]. Yamane et al. showed that AREG expression was augmented in RA tissues [14]. Liu et al. demonstrated that AREG expression was upregulated by IL-1*β* in fibroblast-like synoviocytes in RA, and its overexpression may increase the production of MMP-1 and cadherin-11, which lead to matrix degradation [31]. In addition, AREG plays an essential role in cartilage development, homeostasis, and disease progression. One previous study indicated that EGFR signaling occurs in the suppression of articular cartilage homeostasis and suggested that the activation of EGFR signaling could be a causal factor of OA [32]. The results of our study indicated that AREG protein levels were significantly higher in OASFs than in normal SFs. However, the relationship between AREG in synovial fibroblasts and OA progression is still unclear. In this study, we explored the signaling pathways involved in AREG-induced MMP-13 production in human OA synovial fibroblasts (OASFs). The discovery of AREG/EGFR-mediated signaling pathways increases the understanding of the mechanism of OA pathogenesis, and AREG could be a novel therapeutic intervention strategy for treating OA.

MMPs can break down the extracellular matrices of cells such as collagen and proteoglycans, causing the development of OA [33]. MMPs comprise 23 zinc-containing endoproteinases, including collagenases, gelatinases, stromelysins, matrilysin, metalloelastase, and membrane-type MMPs [34]. MMPs are essential for tissue remodeling, embryogenesis, angiogenesis, and wound healing. MMP-13, a crucial enzyme, degrades collagen types I, II, and III, leading to cartilage breakdown, and exhibits high expression levels in patients with several pathological diseases, such as OA, RA, and cancer [35]. Previous studies have reported that injury causes an increase in MMP-13 expression, and MMP-13 expression is correlated with cartilage degradation in animal models of OA [36]. In addition, one clinical study demonstrated that MMP-13 is not expressed in normal adult cartilage but is highly expressed in the cartilage of patients with OA [37]. We identified MMP-13 as a target protein for the AREG-signaling pathway, which regulated cartilage breakdown. We discovered that AREG induces MMP-13 mRNA and protein expression in a dose- and time-dependent manner in OASFs. We also demonstrated that AREG-induced MMP-13 production requires activation of the EGFR, PI3K, Akt, and NF-*κ*B signaling pathways. These results suggest that AREG acts as an inducer of MMPs and enhances the breakdown of cartilage.

Toulany et al. (2007) reported that the activation of EGFR signaling in the PI3K-Akt pathway plays a vital role in molecular biological functions [38]. Other studies have demonstrated that the EGFR and PI3K/Akt pathways mediate MMP expression and cell invasion in patients with various cancers [39]. The roles of PI3K- and Akt-dependent signaling in the regulation of MMP expression are not consistent across cell types [20]. However, the mechanisms for inducing MMP expression could differ depending on cells type. No studies have examined the signaling pathways that induce MMP expression in OASFs. Our results demonstrated that the treatment of OASFs by using PI3K or Akt inhibitors, or the transfection of cells with PI3K and Akt mutants, reduced AREG-induced MMP-13 expression. However, we also discovered that AREG treatment increased the levels of PI3K and Akt phosphorylation. Moreover, EGFR inhibitors inhibited AREG-mediated PI3K phosphorylation. These results suggest that AREG induced MMP-13 production in the EGFR, PI3K, and Akt signaling pathways in synovial fibroblasts.

Previous research has indicated that NF-*κ*B regulates MMP-13 expression in human chondrocytes [40]. In addition, AREG activates the MAPK and NF-*κ*B pathways in other cells [41]. Therefore, we used NF-*κ*B inhibitors to explore these pathways. Our study demonstrated that NF-*κ*B activation contributed to AREG-induced MMP-13 expression in human synovial fibroblasts. Pretreatment of cells with the NF-*κ*B inhibitors TPCK and PDTC reduced AREG-induced MMP-13 expression. Therefore, the NF-*κ*B binding site is critical in AREG-induced MMP-13 production. The NF-*κ*B sequence binds to members of the p65 and p50 families of transcription factors, and the results of this study indicated that AREG induced p65 phosphorylation and nuclear accumulation. Furthermore, using transient transfection by employing NF-*κ*B-luciferase as an indicator of NF-*κ*B activity demonstrated that AREG increased NF-*κ*B activation. In addition, PD158780, BIBX1382, and PI3K inhibitors (LY294002, Wortmannin), Akt inhibitors (Akti), and PI3K and Akt mutants reduced AREG-increased NF-*κ*B promoter activity. These results indicated that AREG increased NF-*κ*B activation in the EGFR/PI3K/Akt signaling pathway in human OASFs. In conclusion, we explored the signaling pathways involved in AREG-induced MMP-13 production in human synovial fibroblasts. We determined that AREG increases MMP-13 production by binding to the EGFR and activating PI3K/Akt signaling, which enhances NF-*κ*B transcription activity and results in the transactivation of MMP-13 production.

## Supplementary Material

Figure S1: EGF and TGF-alpha attenuate MMP-13 expression in OASFs. (a) OASFs were incubated with EGF (50 ng/ml) and TGF-alpha (50 ng/ml) for 24 h, and cell lysates were then collected. MMP-13 protein levels in cell lysates were determined by Western blot analysis. (n = 4). (b) OASFs were incubated with EGF (50 ng/ml) and TGF-alpha (50 ng/ml) for 24 h. The mRNA expression of MMP-13 was examined by qPCR (n = 4).
Figure S2: AREG slight induced AP-1 activation. OASF were incubated with various concentrations of AREG. AP-1 luciferase activity was measured, and the results were normalized to the *β*-galactosidase activity.
