# Supplementary figures and images for: The Effects of Amphiregulin Induced MMP-13 Production in Human Osteoarthritis Synovial Fibroblast
Source: Mediators Inflamm. 2014 Jul 24;2014:759028. doi: 10.1155/2014/759028 (PMC4131469; doi:10.1155/2014/759028)

Fig. S1

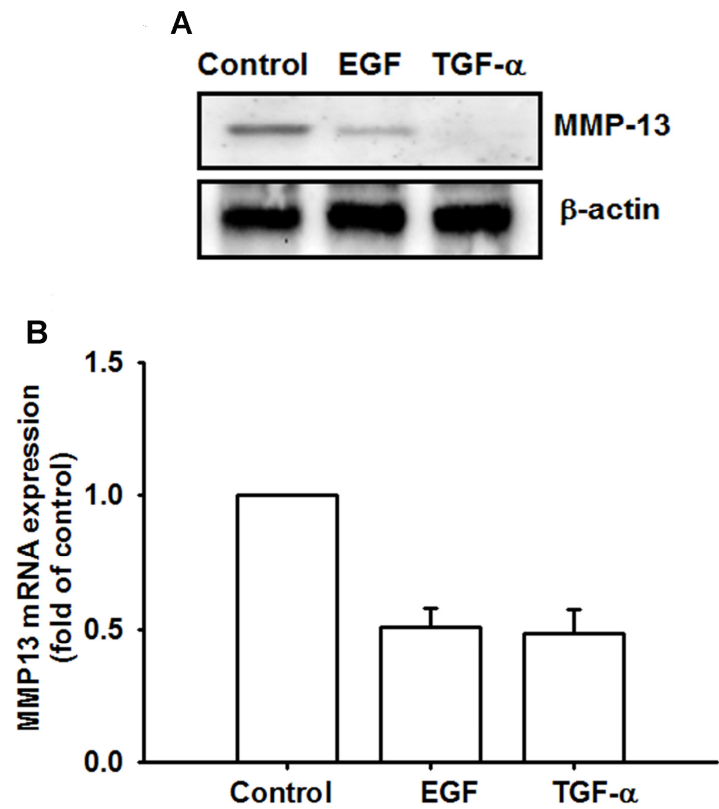

Fig. S2

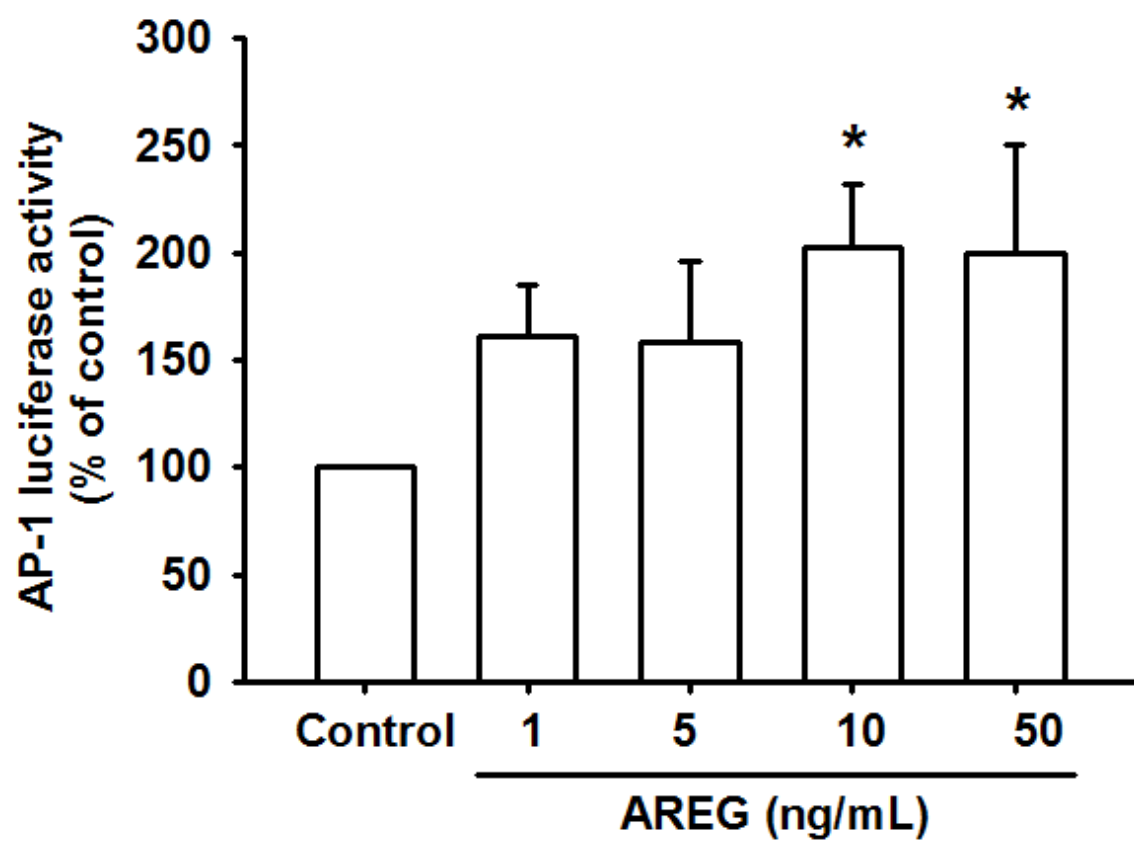

Supplement: Supplementary file 1 — Figure S1: EGF and TGF-alpha attenuate MMP-13 expression in OASFs. (a) OASFs were incubated with EGF (50 ng/ml) and TGF-alpha (50 ng/ml) for 24 h, and cell lysates were then collected. MMP-13 protein levels in cell lysates were determined by Western blot analysis. (n = 4). (b) OASFs were incubated with EGF (50 ng/ml) and TGF-alpha (50 ng/ml) for 24 h. The mRNA expression of MMP-13 was examined by qPCR (n = 4). Figure S2: AREG slight induced AP-1 activation. OASF were incubated with various concentrations of AREG. AP-1 luciferase activity was measured, and the results were normalized to the β-galactosidase activity. [file 759028.f1.pdf]
